# Optical properties of SrF_2_ and SrF_2_:Ce^3+^ crystals codoped with In^3+^

**DOI:** 10.1039/d0ra00865f

**Published:** 2020-04-07

**Authors:** Alexandra Myasnikova, Roman Shendrik, Alexander Bogdanov

**Affiliations:** A.P. Vinogradov Institute of Geochemistry SB RAS Favorski 1A Irkutsk Russian Federation sasham@igc.irk.ru; Irkutsk National Research Technical University Lermontov str. 83 Irkutsk 664074 Russia

## Abstract

The paper presents the results of a comprehensive study of effects that In^3+^ ion impurities have on the optical properties of SrF_2_ and SrF_2_:Ce^3+^ crystals. The investigations were carried out both by optical spectroscopy methods and *ab initio* quantum chemical calculations. To estimate the effect of indium impurities on the optical band gap of the crystals, the DFT calculations were performed for SrF_2_ crystals doped with different concentrations of In^3+^ ions. The study of SrF_2_ crystals co-doped with Ce and In ions reveals the highly effective role of indium ions in reducing the electron trapping efficiency in the processes of excitation transfer to an activator. This improvement is associated with some change in the band gap of the crystals caused by indium doping, which is confirmed by both theoretical and experimental results.

## Abstract 2

In^3+^ states are located close to electron traps and have a band character, the transfer of electrons from conduction band to the luminescence center is possible without significant trapping which facilitates fast luminescence in SrF_2_:Ce^3+^,In^3+^.

## Graphical abstract

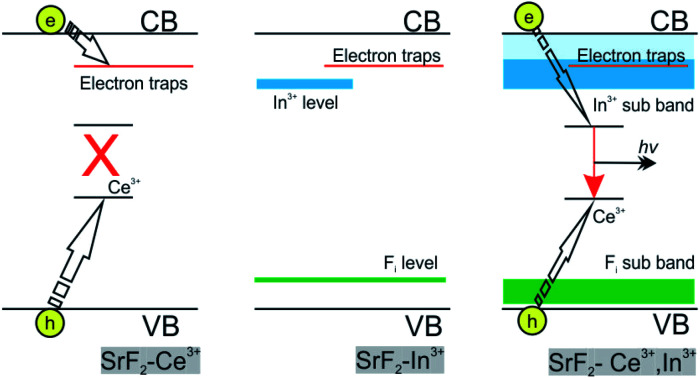

## 1 Introduction

Interest in new scintillation materials contributes to an increasing number of new applications in medicine, science, and national security, which require lower costs and increased production.^1,2^ Commercially available NaI:Tl is among the most popular inorganic scintillators for many applications with a poor energy resolution of at best 6.3% at 662 keV and light output of 44 000 photon per MeV.^3^ However, for some applications (such as well-logging), the temperature stability and the non-hygroscopicity of scintillators are decisive factors. SrF_2_:Ce^3+^ crystal is one of the promising materials for such applications. The light outputs of SrF_2_:0.3%Ce^3+^ crystals measured from X-ray excited luminescence and pulse height spectra were 33 970 and 13 760 photon per MeV respectively.^4,5^ The difference in measured values of light output was explained by the presence of a slow component of the glow, which was associated with shallow electron traps.^4,6,7^

It is well known that the presence of traps adversely affects the scintillation processes in crystals, due to the trapping of electrons and holes at shallow energy levels. Such traps slow down the luminescence process, which worsens the time resolution of the scintillator and its light yield. Therefore, in order to improve the quality of the scintillators, the traps must either be completely eliminated during the growth of the crystal^8^ or their capture cross-section reduced by targeting another structural defect next to the trap (so-called “defect engineering”).^9^

There is another way to get rid of shallow traps – the introduction of a co-activator with a high third ionization potential, which leads to some changes in the band structure of the crystal: band shift, narrowing of the band gap or expansion of the conduction band, depending on the matrix, activator and concentration of the activator. As a result, trap levels fall into the conduction band and do not participate in the energy transfer process. In the last few years, it has been demonstrated that co-doping allows for large improvements in the performance of a variety of scintillator materials.^10–16^ Such a decrease in the influence of shallow traps is well demonstrated when Lu_3_Al_5_O_12_ is doped with the co-activator Ga^3+^ in ref. 17.

In this paper, we present the results of an investigation of the indium ions’ influence on the scintillation properties of strontium fluoride crystals. The investigations were carried out by both optical spectroscopy and DFT quantum chemical calculations. Our study was focused on the SrF_2_ crystals doped with In, but in addition, the results of studies of SrF_2_ with double activation (Ce and In ions) will be presented.

## 2 Methods

### 2.1 Calculations

*Ab initio* calculations of SrF_2_ crystals with indium impurities were carried out within the density functional theory framework using the VASP software complex^18^ with the PBE0 hybrid functional. For calculations, supercells of 2 × 2 × 2 unit cells (in total 96 atoms) were constructed, and one, two, three or four divalent alkaline-earth atoms were replaced by In atoms. In fluorite-type crystals, indium ions mainly enter the trivalent state.^19,20^ Since no additional impurities other than indium and cerium are doped during the crystal growth, there are two possible ways of forming excess charge compensator.^21^ It can be interstitial fluorine ions or oxygen ion O^2−^, which is formed at the site of the anion. The latter are an undesirable effect and their presence is strictly controlled by luminescent methods.^22^ Therefore, in this work, we will consider only interstitial fluorine ions as charge compensators. It is known that interstitial fluoride ions in alkaline-earth fluorides can form centers of *C*_3v_, *C*_4v_ or *O*_h_ symmetry with respect to the rare-earth ion.^23^ We examined all these cases of defect symmetry, but no significant difference was found in the calculations. Hereafter we will consider the charge compensation of the trivalent In^3+^ ion as an interstitial fluorine atom put in the *C*_4v_ symmetry position near the impurity. The positions of the atoms and the symmetry of the crystal used in the calculations were taken from the ref. 24 and 25. The calculations were carried out both for defect-free crystals and for crystals containing 1, 2, or 4 indium ions. This corresponds to an impurity concentration of 2.1, 4.2 or 8.3 mol% In^3+^, respectively. Geometry optimization was performed within the framework of the generalized gradient approximation (GGA) using the PBEsol exchange–correlation functional.^26^ The shape of the supercells was kept fixed during optimization. A *G*-centered regular Monkhorst–Park *k*-points mesh of 3 × 3 × 3 with 8 *k*-points in the irreducible Brillouin zone has been used for the calculations. The convergence was considered achieved if the difference in total energies between the two last iterations did not exceed 10^−5^ eV. Lattice relaxation was considered achieved when the largest value of interatomic forces was smaller than 10^−6^ eV Å^−1^.

As a result of geometry optimization, we obtained a lattice constant for strontium fluoride equal to 5.44 Å, which is somewhat lower than that calculated by the localized density approximation (LDA) method in ref. 24 (5.6 Å) and the experimental value of ref. 25 (5.79 Å). Nevertheless, we considered these data satisfactory for our purposes, since it is important for us only to track changes in the band structure with the indium impurity doping. DOS (density of states) calculations were performed using a hybrid functional PBE0 at the gamma point. The band gap of the SrF_2_ crystal, estimated as the energy difference between the highest occupied molecule orbital (HOMO) and the lowest unoccupied molecule orbital (LUMO), is 10.9 eV for calculations using the hybrid functional PBE0. This value is also slightly lower than the experimental band gap (11.2 eV (ref. 27)). For more accurate calculations of the band gap, it is necessary to use more resource-expensive methods such as GW.^28^ However, we did not pose such a problem and consider the obtained accuracy acceptable for our calculations.

### 2.2 Experimental

Oxygen-free SrF_2_ crystals both pure and doped with different concentrations of cerium and indium were grown in a graphite crucible by the Stockbarger method. Electron probe microanalysis was performed on carbon-coated strontium fluoride single crystals doped with preliminary dehydrated InF_3_ to determine the chemical composition of the samples. A JEOL JXA-8200 electron probe microanalyzer operated at an accelerating voltage of 15 kV and a beam current of 5 nA was used (spot size 1 μm and counting time 40 s). Full wavelength dispersive spectrometry (WDS) mode was employed. The average compositions (determined over eight spots) are Sr_0.94_F_2_In_0.06_ for SrF_2_-7 mol% InF_3_;Sr_0.98_F_2_In_0.02_ for SrF_2_-3 mol% InF_3_ and Sr_0.99_F_2_In_0.01_ for SrF_2_-1 mol% InF_3_ samples. The cerium concentrations in the growing crystals were determined by the method of inductively coupled plasma mass spectrometry (ICP-MS) from a solution obtained after dissolving the crystal and separating strontium as the main interfering element.^29,36^ The concentration of Ce ions in the investigated crystals was about 0.09 mol%. It should be noted that these values indicate the impurity concentrations introduced during crystal growth.

Scintillation decay time curves under ^137^Cs *E* = 662 keV gamma source excitation were recorded by a 200 MHz oscilloscope Rigol DS-1202CA. To register decay curves in wide time intervals, we used an oscilloscope input resistance set at 50 Ω and 2.8 kΩ.^4^ Absorption spectra in the vacuum ultraviolet (VUV) spectral region were measured using a VMR2 monochromator and a deuterium lamp Hamamatsu L7292 as the VUV source. Registration was performed with a solar-blind photomultiplier FEU-142. A sample was mounted in a vacuum cryostat. A type-K thermocouple with cold end compensator at 77 K was used to control the temperature. Thermally stimulated luminescence (TSL) was detected with a grating monochromator MDR-2 and a photomodule Hamamatsu H6780-04. TSL glow curves were measured after irradiation of crystals at 77 K during linear heating at a 20 K min^−1^ rate. X-ray excited and thermally stimulated luminescence was performed using an X-ray tube with a Pd anode operating at 50 kV and 1 mA. The spectra were recorded in the photon-counting regime using a photomodule Hamamatsu H6780-04. For comparison of intensity the size and shape of the samples was the same. The thickness of the samples was 1.5 mm. All samples were sawed and one side polished. The methodology of sample comparison was given in ref. 4, 6 and 30.

## 3 Results and discussion

### 3.1 Calculation

**Fig. 1:**
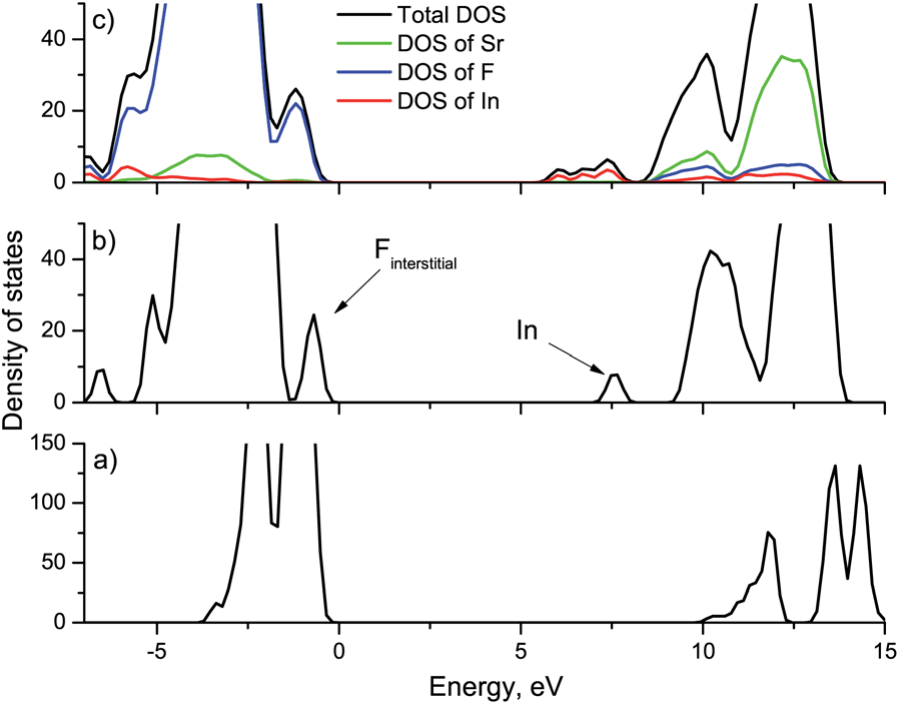
Calculated density of states for different concentrations of In^3+^ doping: (a) SrF_2_ crystal, (b) SrF_2_ crystal with 2 indium ions, (c) SrF_2_ crystal with 4 indium ions. Calculations were performed within the PBE0 hybrid functional.

To study the effect of indium ions on the band gap of the crystals, calculations were performed with different concentrations of impurity ions. Fig. 1 shows the calculated density of states (DOS) for strontium fluoride crystals with different indium concentrations, as well as the partial density of states. In a defect-free strontium fluoride crystal, the valence band is formed from 2p orbitals of fluorine ions, and the conduction band is formed by 5s orbitals of strontium ions. The electronic configuration of the In^3+^ ion is [Kr]4d^10^5s^0^5p^0^. So, in SrF_2_ crystals doped with indium ions, an indium level is formed corresponding to 5s orbitals near the conduction band of the crystal. In addition, the upper occupied indium 5d orbitals become part of the valence band of the crystal. As already mentioned above, to compensate for the excess charge of the trivalent impurity, we consider an additional interstitial fluorine ion, since the crystal as a whole must be electrically neutral. Interstitial fluorine ions (F_i_^−^) in alkaline earth fluoride crystals create levels near the top of the valence band corresponding to their 2p orbitals.^31^ The calculation results show that in our case a sub-band is also created in the conduction band corresponding to interstitial fluorine ions (marked by an arrow in Fig. 1(b)). With increasing concentration, the impurity levels in the band gap will create sub-bands formed at the bottom of the conduction band and near the top of the valence band (Fig. 1(c)). Interstitial fluorine ions are also formed in SrF_2_–Ce^3+^ crystals, however, as a rule, they have no effect on energy transfer processes. Therefore, the effect of the formation of a sub-band of interstitial fluorine ions will not be discussed further. In this case, we cannot say that there is an expansion of the conduction band and a decrease in the energy band gap. However, it can be said that upon doping with indium, the band structure of the crystal changes, which should affect its optical characteristics. In particular, the sub-band of indium ions can participate in absorption, while the vibrational path of excitation transfer to the conduction band is possible. Note that our calculations do not include the vibrational component and all calculations are performed for absolute zero, while optical measurements will be carried out at room temperature. According to the calculated data (Fig. 1(c)), the indium sub-band is approximately 1 eV away from the bottom of the conduction band with a sub-band width of 1.4 eV for 4 In ions in the lattice (which corresponds to 8.3 mol% In^3+^). Thus, it can be said that most electron traps with an activation energy of less than 2 eV are most likely to fall in energy into the indium sub-band and cease to participate in energy transfer processes.

### 3.2 Experimental

**Fig. 2:**
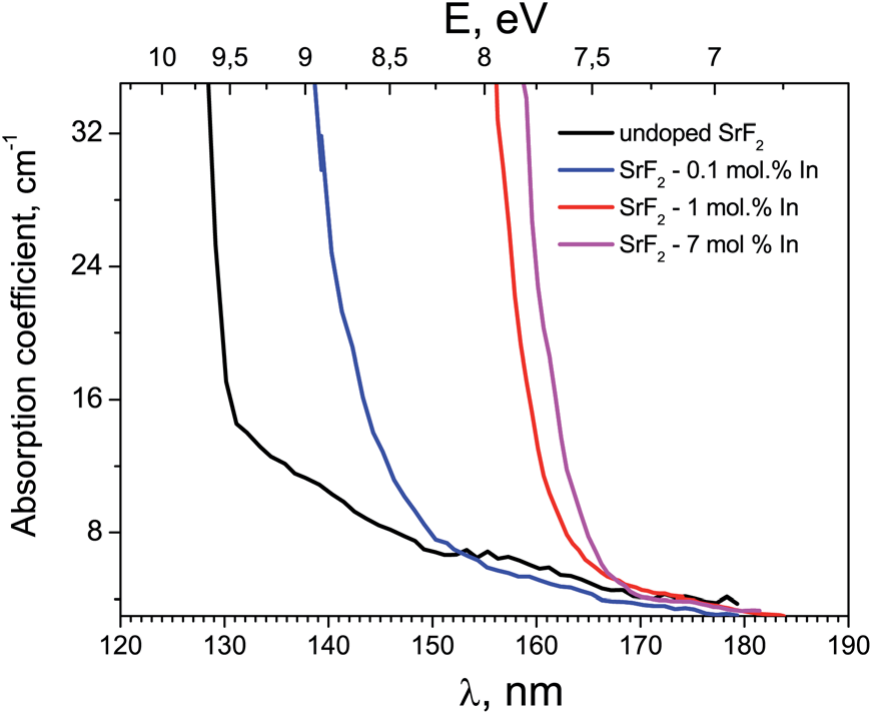
Optical absorption spectra of indium doped SrF_2_ crystals.

Fig. 2 shows the absorption spectra of SrF_2_ crystals with different concentrations of indium ions in the fundamental absorption spectral region. It can be seen that an increase in the impurity concentration leads to a shift the fundamental absorption of the crystals to the low-energy side. That is, we can speak of a decrease in the band gap of the crystal with an increase in the indium concentration. Note that hereafter we speak more about the optical band gap, since it is clear that the doping with a small amount of impurity does not substantially change the band gap itself. This shift in fundamental absorption is in good agreement with the conclusions of Section 3.1, in particular, it confirms at least the formation of a sub-band near the bottom of the conduction band associated with indium ions. In this case, with an increase in the indium concentration, this sub-band expands and its participation in interband transitions is more efficient.

**Fig. 3:**
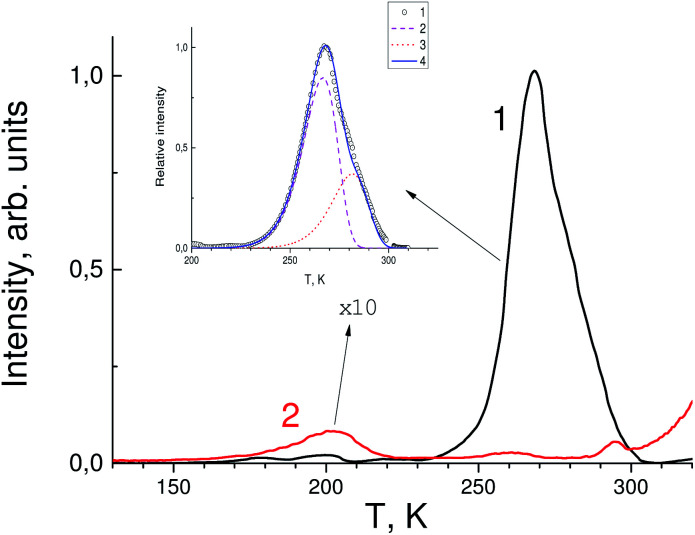
Thermally stimulated luminescence glow curves of SrF_2_-0.3 mol% Ce^3+^ (black curve 1) and SrF_2_-0.3 mol% Ce^3+^ and 3% In^3+^ single crystals (red curve 2). The intensity of the red curve is increased tenfold. Inset: the glow peak at 270 K (circles), results of the peak deconvolution (curves 2 and 3) and total calculated curve using a Randall–Wilkins equation (4).^38^

Fig. 3 shows the TSL measurements of both SrF_2_ crystals doped with cerium ions and co-doped with cerium and indium ions. It is known that hole centers (V_k_) are created in alkaline-earth fluoride crystals with impurities of trivalent ions, the most stable of which are those located at interstitial ions (V_kH_) or near impurity ions (V_kA_).^32–35^ The latter are the most stable up to room temperature.^7,32,36,37^ In the TSL glow curves in Fig. 3, low intensity bands associated with V_kA_ hole centers near 160–200 K are also observed. However, in the TSL spectra of the SrF_2_–Ce^3+^ crystal, a more intense peak is observed at 270 K. This peak can be associated precisely with electronic traps. In only the Ce^3+^-doped sample complex TSL is the peak at 270 K observed. The depth of traps attributed to this peak was determined using two different TSL methods. The first method is using a simple equation:

1 *E* = 2.52*k*_B_*T*_m_^2^/FWHM − 2*k*_B_*T*_m_

with *E* the trap depth in eV, *k*_B_ is Boltzmann constant (eV K^−1^), *T*_m_ is the temperature (K) of the glow peak. The trap depth in this case is about 0.63 eV. In the second method the whole measured glow curve is fitted by a mathematical description of the glow peak using a first-order kinetic Randall–Wilkins equation.^38^ It assumes that the charge transfer can be described by first-order kinetics and the trap can be characterized by two trapping parameters, *s* (s^−1^), a frequency factor and the trap depth or activation energy *E* (eV). The glow peak has a complex origin and it is deconvoluted into two peaks corresponding two kinds of traps with *E* = 0.69 eV and 0.73 eV and *s* = 1 × 10^−12^ s^−1^ (inset in Fig. 3). The depth of the traps is less than the shift of the fundamental absorption edge and, correspondingly, the decrease in the band gap in In^3+^-codoped crystals. Therefore, such traps are not able to capture electrons and the TSL peak in the crystals SrF_2_–Ce,In disappears.

After In^3+^ co-doping its intensity is decreased by more than one order of magnitude. This is most likely due to the fact that the levels of traps responsible for the TSL peak fall into the sub-band formed by indium orbitals near the bottom of the conduction band. Nevertheless, the peak at 270 K has a complex nature and is associated not only with electronic traps, whose contribution almost disappears after doping with indium, but also with hole centers that are not affected by indium activation. The paper ref. 39 shows that there are some centers in SrF_2_–Ce^3+^ crystals that are destroyed at a temperature close to 270 K, which correlates well with our TSL data. It can be assumed that the shallow traps are photochromic centers (PC) which consist of a rare-earth ion and a vacancy that has captured an electron (F center). According to calculations performed for CaF_2_–Lu^3+^ crystals,^40^ the levels of photochromic centers lie near the bottom of the conduction band and may well fall into the sub-band formed by impurity indium ions.

Electron traps in alkaline-earth fluoride crystals doped with trivalent ions also influence the luminescence kinetics. This was first shown in ref. 7, where the slow component of the cerium luminescence was found. Further, in ref. 4, the scintillation decay time of strontium fluoride crystals doped with Ce^3+^ was analyzed in more detail. In addition, it was found that for several experimental techniques, different values of the light output of the SrF_2_–Ce^3+^ crystal were obtained. All these facts are taken into account within the electron traps model, the presence of which leads to an increase in the lifetime of the excited state of the cerium ion and delays the decay time. Thus, we can expect a change in the kinetics of luminescence if our prediction about the effect of indium impurities on the number of electron traps is true.

**Fig. 4:**
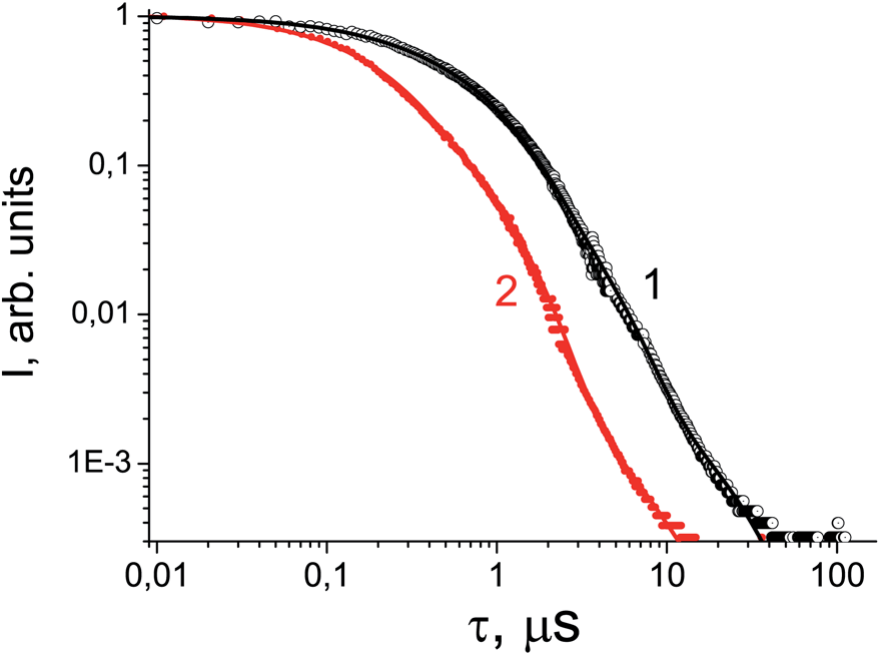
Luminescence decay curve under ^137^Cs excitation of SrF_2_-0.1 mol% Ce^3+^ (circles, curve 1) and SrF_2_-0.1 mol% Ce^3+^,3% In^3+^ (dots, curve 2).

**Fig. 5:**
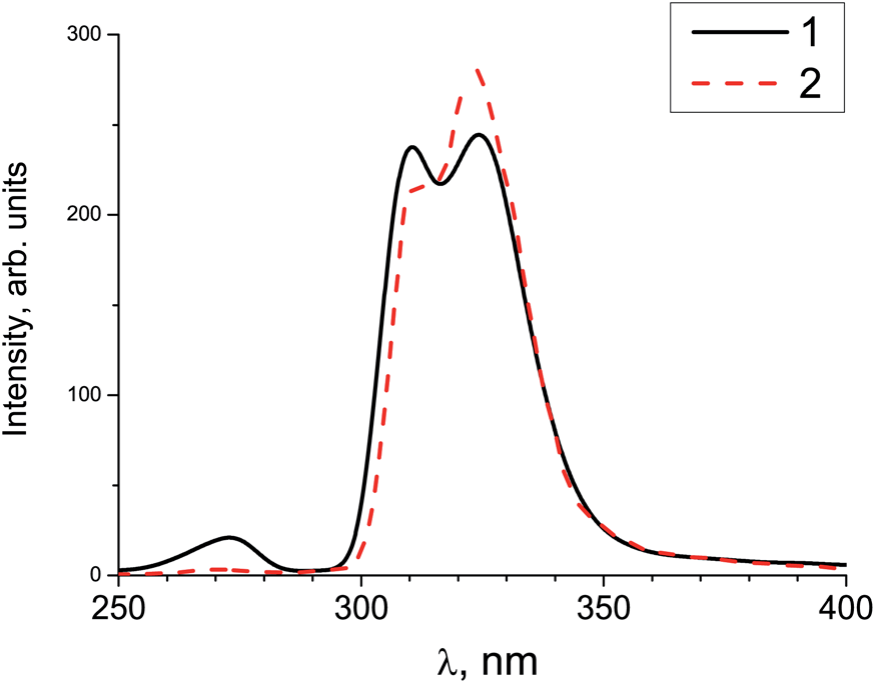
X-ray excited luminescence of SrF_2_-0.1 mol% Ce^3+^ (solid curve 1) and SrF_2_-0.1 mol% Ce^3+^,3% In^3+^ (dashed curve 2) measured at 300 K.

Gamma-ray excited luminescence decay curves are shown in Fig. 4. It is clearly seen that co-doping with In^3+^ ions leads to decreasing contributions of longer components to the luminescence decay. Under direct excitation of the 4f–5d band at about 280 nm single exponential luminescence decay is observed. The decay time constant is about 30 ns.^30^ However, under X-ray excitation several components in the decay time profile appear. The shortest component at about 180 ns could be ascribed to the fast resonance energy transfer in nearest pairs of self-trapped excitons and cerium ions.^41^ Longer stages in the scintillation time profile can be attributed to thermo-activated processes related to electron or hole delayed transfer to the activator ion. A similar energy transfer mechanism has been observed in SrF_2_ doped with Pr.^36^ However, the intensity of X-ray excited luminescence is equal in SrF_2_-0.1% Ce^3+^ and SrF_2_-0.1% Ce^3+^,3% In^3+^ samples (Fig. 5). The most intense bands at 310 and 325 nm in the X-ray luminescence spectra correspond to the 5d–4f emission of Ce^3+^ ions. The wide band peaked at 270 nm is attributed to self-trapped exciton luminescence.^42^ This fact can be explained by the band gap change in SrF_2_–Ce^3+^;In^3+^ relative to SrF_2_–Ce^3+^ crystals and, consequently, the reduced role of shallow traps in the scintillation energy transfer process.^4^

According to the calculated and experimental data the effect of In^3+^ co-doping on the luminescence properties of Ce^3+^-doped SrF_2_ crystals consists in the forming of an In^3+^ sub-band within the band gap of the host crystal which facilitates electron transfer from the conduction band to the luminescence center without trapping on intrinsic defects. The electronic traps in SrF_2_ occur 0.7–0.8 eV (ref. 30 and 37) below the conduction band. These traps catch electrons from the conduction band and slow down their transfer to the Ce^3+^ luminescence center. However, the introduction of In^3+^ ions produces unoccupied electronic levels near 2.0 eV below the conduction band. When the amount of In^3+^ doping increases, the corresponding localized states adopt a band character due to wave-function overlapping. We assume that overlapping between shallow electron traps and the indium band also occurs. Thus the role of the electron traps becomes negligible as trapped electrons can easily move to the In^3+^ band. Now, because In^3+^ states are located close to electron traps and have a band character, the transfer of electrons from the conduction band to the luminescence center is possible without significant trapping which facilitates fast luminescence.

## 4 Conclusions

The work presents study of band gap behavior in In^3+^ doped SrF_2_ crystals, carried out both by *ab initio* methods in terms of the density functional approach and using optical spectroscopy methods. DFT calculations showed that the indium co-doping of SrF_2_ crystals leads to the formation of a sub-band corresponding to 5s orbitals of In^3+^ near the conduction band of the crystal. Absorption spectra also show a shift in fundamental absorption, which increases with increasing impurity concentration. This fact confirms the calculated data on the change in the structure of the band gap of the crystal upon doping with indium. Study of thermoluminescence and the decay time of SrF_2_–Ce^3+^ and SrF_2_–Ce^3+^,In^3+^ crystals has shown that the doping with indium leads to a noticeable decrease in the role of electron traps in the glow processes. Thus, indium doping as a co-activator to alkaline-earth fluoride crystals doped with rare-earth ions can improve the scintillation properties of the latter, due to a decrease in the concentration of shallow traps formed in the band gap of crystals.

## Conflicts of interest

There are no conflicts to declare.

## Supplementary Material
